# A Novel Lamin A Mutant Responsible for Congenital Muscular Dystrophy Causes Distinct Abnormalities of the Cell Nucleus

**DOI:** 10.1371/journal.pone.0169189

**Published:** 2017-01-26

**Authors:** Alice Barateau, Nathalie Vadrot, Patrick Vicart, Ana Ferreiro, Michèle Mayer, Delphine Héron, Corinne Vigouroux, Brigitte Buendia

**Affiliations:** 1 Unité de Biologie Fonctionnelle et Adaptative (BFA), CNRS UMR 8251, Université Paris Diderot, Sorbonne Paris Cité, Paris, France; 2 Centre de Référence des Maladies Neuromusculaires de l’Est Parisien, Secteur Pédiatrique, Hôpital d’Enfants Armand Trousseau, Paris, France; 3 Centre de Référence Déficiences Intellectuelles, Département de Génétique et INSERM U975, Groupe hospitalier Pitié-Salpétrière, Paris, France; 4 Sorbonne Universités, UPMC Univ Paris 6, Inserm UMR_S938, Centre de Recherche Saint-Antoine, Institute of Cardiometabolism and Nutrition (ICAN), AP-HP, Hôpital Saint-Antoine, Laboratoire Commun de Biologie et Génétique Moléculaires et Service d’Endocrinologie Diabétologie et Endocrinologie de la Reproduction, Paris, France; Rutgers University Newark, UNITED STATES

## Abstract

A-type lamins, the intermediate filament proteins participating in nuclear structure and function, are encoded by *LMNA*. *LMNA* mutations can lead to laminopathies such as lipodystrophies, premature aging syndromes (progeria) and muscular dystrophies. Here, we identified a novel heterozygous *LMNA* p.R388P de novo mutation in a patient with a non-previously described severe phenotype comprising congenital muscular dystrophy (L-CMD) and lipodystrophy. In culture, the patient’s skin fibroblasts entered prematurely into senescence, and some nuclei showed a lamina honeycomb pattern. C2C12 myoblasts were transfected with a construct carrying the patient’s mutation; R388P-lamin A (LA) predominantly accumulated within the nucleoplasm and was depleted at the nuclear periphery, altering the anchorage of the inner nuclear membrane protein emerin and the nucleoplasmic protein LAP2-alpha. The mutant LA triggered a frequent and severe nuclear dysmorphy that occurred independently of prelamin A processing, as well as increased histone H3K9 acetylation. Nuclear dysmorphy was not significantly improved when transfected cells were treated with drugs disrupting microtubules or actin filaments or modifying the global histone acetylation pattern. Therefore, releasing any force exerted at the nuclear envelope by the cytoskeleton or chromatin did not rescue nuclear shape, in contrast to what was previously shown in Hutchinson-Gilford progeria due to other *LMNA* mutations. Our results point to the specific cytotoxic effect of the R388P-lamin A mutant, which is clinically related to a rare and severe multisystemic laminopathy phenotype.

## Introduction

The *LMNA* gene encodes A-type lamins, intermediate filament proteins that are regulated during development and cell differentiation. The two major isoforms in mammals are lamins A and C (LA/C). Prelamin A (preLA, 664 amino acids (a.a.)) and lamin C (LC, 572 a.a.) are produced by alternative splicing. Mature lamin A (mLA, 646 a.a.) is generated after posttranslational modifications of prelamin A, a process which includes farnesylation and carboxymethylation of Cysteine 661 followed by proteolysis of the last 18 a.a. by the metalloproteinase Zmpste24 [1]. In cells, A-type lamins form a lamina network at the nuclear envelope (NE) and a pool in the nuclear interior. By binding numerous partners (the LINC complex, inner nuclear membrane proteins, factors involved in gene expression regulation, and DNA), A-type lamins modulate nuclear shape, nuclear mechanics, genome integrity, transcriptional events and signal transduction [2].

In the last 15 years, ~400 mutations in the *LMNA* gene have been identified as causal for laminopathies, which encompass tissue-specific dystrophies affecting cardiac, cartilage, bone, skin, peripheral nerves, skeletal muscles and/or adipose tissues. Laminopathies include muscular dystrophies and/or cardiomyopathies, lipodystrophies and syndromes of premature ageing such as Hutchinson-Gilford progeria syndrome (HGPS) [3, 4].

Wild-type lamin A helps regulate the normal ovoid nuclear shape within cells. Accordingly, the extinction of lamin A/C by siRNA or the expression of laminopathy-associated mutant lamins A/C induces abnormal nuclear shapes [5, 6]. For instance, abnormal nuclear blebbing was reported for subpopulations of patient fibroblasts in the context of lipodystrophies [7, 8], autosomal dominant Emery-Dreifuss muscular dystrophy (AD-EDMD) [9, 10] and dilated cardiomyopathy (DCM) [11]. Additionally, the formation of nuclear lobulations has been documented for progeroid syndromes and HGPS [12–15]. Although nuclear dysmorphy is frequently associated with laminopathies, this criterion is not sufficient to distinguish one laminopathy from another. In addition, nuclear dysmorphy originates through diverse mechanisms including changes in i) nucleocytoskeletal interactions, ii) nuclear membrane composition, and/or iii) chromatin conformation [16]. Understanding the underlying mechanisms is relevant to preventing or reversing nuclear dysmorphy, including for therapeutic purposes, since an abnormal nuclear shape impedes nuclear function [8]. Under *LMNA* inhibition or in HGPS, the frequency of nuclear dysmorphy can be reduced by relieving the forces exerted on the nuclei by microtubules, using drugs such as nocodazole or an N-acetyl transferase 10 inhibitor [17], and also by reducing the accumulation of SUN1, a protein of the LINC complex that connects the inner nuclear membrane with the cytoskeleton [18]. In addition, treatment with farnesylation inhibitors, by reducing the amount of the truncated, constitutively farnesylated prelamin A (called progerin) responsible for HGPS, improves nuclear shapes at the cellular level [19], ameliorates some aging-like phenotypes at the clinical level and increases longevity in mice and humans with HGPS [20, 21].

We describe here a patient with a severe phenotype, congenital muscular dystrophy (L-CMD) and lipodystrophy, in whom we detected a novel heterozygous *LMNA* p.R388P de novo mutation. In contrast to the previous studies, our study revealed that nuclear dysmorphy associated with the R388P lamin A mutation responsible for congenital muscular dystrophy L-CMD and lipodystrophy relies on distinct mechanisms.

## Materials & Methods

### *LMNA* gene sequencing

Clinical, biological and molecular studies of the patient and her relatives (parents and her healthy sister) were performed after full informed consent according to legal procedures. Genomic DNA was extracted from peripheral-blood leukocytes using a commercial kit (FlexiGene DNA Kit, Qiagen). The entire coding region and splice junctions of *LMNA* were amplified by PCR with specific primers in the proband and her relatives for direct sequencing as previously described [22]. Direct Sanger sequencing of PCR fragments was performed using the ABI dye terminator mix on an Applied Biosystems 373A DNA sequencer (Perkin Elmer).

### Primary cultures of skin fibroblasts

Subcutaneous fibroblasts obtained via skin biopsy from the patient (aged 16) and from two healthy individuals (her 43-year-old mother and an unrelated 18-year-old man) were cultured in low-glucose DMEM containing GlutaMAX^™^ and pyruvate (Gibco), and 10% foetal calf serum for experimental use between passages 6 and 14. We are authorised to store and use our registered and anonymised collection of human samples in the INSERM Unit number 938 (Centre de Recherche Saint-Antoine) (anonymised collection registered under number DC-2008-529). We obtained specific authorisation for the study “Physiopathological modelling of insulin resistance and/or lipodystrophic syndromes” from the ethics committee "Comité de Protection des Personnes Ile-de-France V" (session of the 3rd March 2015, examination of project b-3-15). In accordance, a written consent was obtained from the patient, her legal representative, and controls subjects for their participation in this study and its potential publications.

Whole cell extracts of fibroblasts from controls and proband were analysed by western blot using antibodies directed against lamins (rabbit anti-lamin A/C and rabbit anti-lamin B1 as described in [7, 23]) and GAPDH (rabbit anti-GAPDH, Sigma-Aldrich).

### Beta-galactosidase senescence assay

Fibroblasts adherent to coverslips were fixed for 12 min at 22°C with 3% formaldehyde, then incubated overnight at 37°C in 1 mg/mL X-gal, 40 mM citric acid, 126 mM sodium phosphate pH 6.0, 5 mM potassium ferricyanide, 5 mM potassium ferrocyanide, 150 mM NaCl, and 2 mM MgCl_2_. Senescence-associated- (SA) β-galactosidase (β-gal) activity was estimated by quantifying the percentage of positive blue cells by direct observation of the samples under light microscope (n = 900–1100 cells in each case).

### C2C12 cell culture and transfection

C2C12 cells were obtained from American Type Culture Collection and grown in DMEM containing high glucose and GlutaMAX^™^ (Gibco) and 10% foetal bovine serum. For cell transfection, lipofectamine^™^ 2000 (Life technologies) was used according to the manufacturer's instructions. Cells were analysed 24 h after transfection.

### Plasmids

Constructs encoding FLAG fusions of WT or p.L647R prelamin A (a.a. 1–664) and mature lamin A (a.a. 1–646) were generated by PCR amplification of the relevant regions of cDNA using as templates pSVK3-prelamin A and pSVK3-mature-lamin A [24]; amplified DNA was ligated into the *Bam*H1 and *Eco*R1 sites of pCMV-Tag 2A (Stratagene). The construct encoding GFP fusion of WT prelamin A was previously described [23]. Vectors encoding lamin A with the p.R388P mutation (pEGFP-R388P-preLA, pCMV-R388P-FLAG-preLA, pCMV-R388P-L647R-FLAG-preLA, pCMV-R388P-FLAG-mLA) were constructed by site-directed mutagenesis using the QuickChange Lightning site-directed mutagenesis kit (Agilent Technologies) with the following primers: forward 5’ GAG GAG AGG CTA CCA CTG TCC CCC AGC 3’ and reverse 5’ GCT GGG GGA CAG TGG TAG CCT CTC CTC 3’.

### Drug treatments of cells

Six hours after transfection, cells were incubated in the presence of 10 μM mevinolin (Sigma-Aldrich) or in 1% DMSO for 18 h. Twenty-four hours after transfection, cells were incubated with a cytoskeleton destabilising drug: nocodazole (Sigma-Aldrich) at 5 or 10 μM for 3 h, or cytochalasin D (Sigma-Aldrich) at 1 μM or 2 μM for 3 h. Alternatively, cells were incubated in the presence of either trichostatin A (TSA, Sigma-Aldrich) at 100 nM or anacardic acid at 100 μM for 16 h.

### Antibodies and immunological methods

For immunofluorescence (IF), cells were fixed with a 3% paraformaldehyde solution (PFA), then processed as previously described [23]. IF was observed under confocal microscopy (Zeiss LSM 700) in the imaging facility of the BFA Institute. Primary antibodies used for IF and immunoblotting (Iblot) analysis were rabbit anti-lamin A/C (as described in [7, 23]), mouse anti-lamin A/C (Novocastra), goat anti-prelamin A (Santa Cruz Biotochnology), rabbit anti-LAP2α (Immuquest, UK), mouse anti-emerin (NCL, Novocastra), rabbit anti-FLAG (Sigma-Aldrich), mouse anti-FLAG (Sigma-Aldrich), mouse anti-GFP (Roche), rabbit anti-GAPDH (Sigma-Aldrich), mouse anti-actin (Millipore), mouse anti-desmin (RD301, Santacruz), mouse anti-α-tubulin (DM1A, Sigma), rabbit anti H3K9ac (06–942, Upstate) and rabbit anti-H3K27ac (ab4729, Abcam).

### Nuclear dysmorphy evaluation

Nuclear dysmorphy was estimated from FLAG IF images, either directly by visual observation or indirectly when specified using Image J software to calculate the nuclear circularity (4π x area / perimeter^2^; a form factor of 1 representing a perfect circle).

### In situ detection of protein-protein interactions by proximity ligation assay (PLA)

In situ protein-protein interactions were detected by PLA [25] in fixed C2C12 cells expressing WT or R388P FLAG-LA. Duolink PLA probe anti-rabbit plus, Duolink PLA probe anti-mouse minus and Duolink detection reagents orange (detected with a Cy3 filter) were used according to manufacturer’s instructions (Olink Bioscience). Detection of FLAG-transfected cells also required the addition of Cy2-conjugated secondary goat antibodies against rabbit. Imaging was performed by confocal microscopy as above. Quantitative analysis of PLA signals was performed using a custom image recognition Image J plugin that relied on a two-step algorithm, as described previously [26]. Data were compiled as the intensity of PLA signals as a function of their distance to the closest point of the nuclear periphery. Considering the distance between the periphery and the nucleus barycentre as the maximal distance (100%), the peripheral signals refer to those detected within an intranuclear peripheral region limited to 10% of the maximal distance.

### Protein extract preparation and protein analysis by western blot

Whole cell extracts were prepared by directly resuspending cells in Laemmli sample buffer. In some experiments, cell fractionation was performed following a procedure adapted from Fey and colleagues [27] and briefly described previously [23]. Protein extracts were analysed by immunoblot as described [26]. Gel quantification was performed by using ImageJ software.

### Statistical analysis

Quantitative results are expressed as means ± standard error of the mean (s.e.m.), or medians with third and first quartiles. Comparisons between samples were performed with the Mann-Whitney test, the Kruskal-Wallis test with or without the pairwise comparisons of groups or the two-way ANOVA test coupled with the Bonferroni post-tests, as indicated.

## Results

### Clinical phenotype

A female patient born from healthy unrelated Caucasian parents presented with unstable gait and frequent falls since gait acquisition at age 12 months, later associated with difficulties climbing stairs, inability to run and limited walking distance. Examination at age 3 years showed waddling gait with marked hyperlordosis, limb girdle muscle weakness predominating in pelvic girdle, calf hypertrophy and arreflexia. Creatine phosphokinase (CPK) level was elevated (up to 1951 UI/L), electromyography was myopathic, brain magnetic resonance imaging and Fukutin-related protein gene (FKRP) sequencing were normal and a muscle biopsy showed dystrophic changes (with normal expression of merosin, dystrophin, alpha and beta-sarcoglycans, calpain and dysferlin). The course of disease was steadily progressive, leading to loss of independent ambulation outdoors at 5 years, joint contractures (spine, right equinovarus foot) from age 6.5 years that initially coexisted with knee hyperextension (genu recurvatum) requiring casts and/or braces and, subsequently, total loss of ambulation and generalised joint contractures (rigid spine, elbow, knee, hips, ankles) from 11 years. Dorsal hyperlordosis was associated with restrictive respiratory insufficiency that appeared at 9 years and was slowly progressive (Forced Vital Capacity (FVC) = 49% of the predicted value and moderate polysomnography abnormalities at 18 years). Holter studies revealed symptomatic supraventricular hyperexcitability at age 10 years and mixed (supra- and ventricular) symptomatic hyperexcitability from 12 years with ventricular doublets and extrasystoles, which were improved by treatment with bisoprolol. Systolic function was normal. No conduction block was observed on 24-h holter-electrocardiogram, regularly performed every 6 months. In addition, a lipodystrophic morphotype developed progressively from early puberty (age 10), with a striking accumulation of adipose tissue in the cervicofacial and subclavicular regions, and a subcutaneous lipoatrophy of distal upper and lower limbs, while BMI remained in the normal range (17.1 kg/m^2^ at age 11, 60th percentile). Glucose tolerance abnormalities, hyperinsulinemia and oligomenorrhea improved with dietary management while lipodystrophy persisted. Serum triglycerides were normal. A c.1163G>C heterozygous change in *LMNA* exon 7, predicting a p.R388P substitution, was identified in the patient (at age 11, la Pitié-Salpétrière Molecular Biology Department, Assistance Publique-Hôpitaux de Paris, France) but not in her parents or her healthy sister. This de novo mutation was absent in more than 150 unrelated control subjects. Sequence alignment of the mutated region across various species revealed that residue p.R388 is highly conserved during evolution (S1 Fig). The *LMNA* p.R388P variation was not found in ExAC or 1000G databases and was predicted to be damaging by PolyPhen-2, SIFT and Mutation Taster.

### Skin fibroblasts of the patient bearing the *LMNA* p.R388P mutation enter prematurely into senescence and show defects in lamina organisation

**Fig 1:**
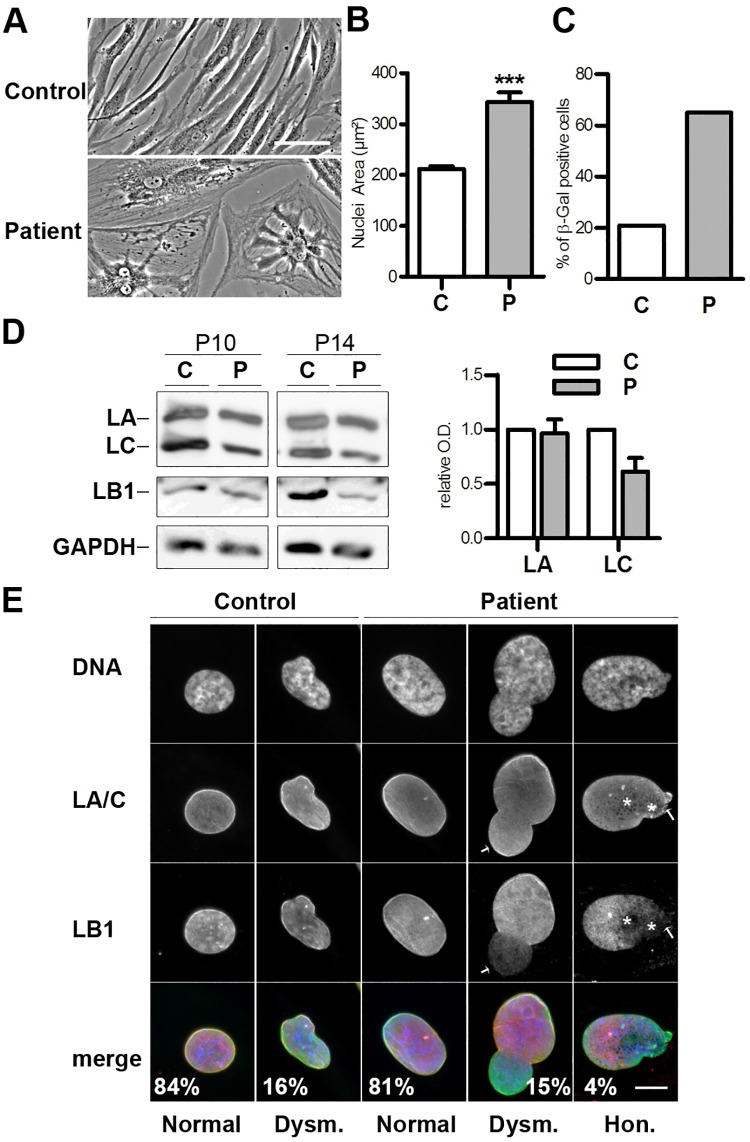
Abnormal cellular phenotypes of fibroblasts from the patient with *LMNA* p.R388P-associated L-CMD and lipodystrophy. **A)** Phase-contrast images of control (passage 11) and patient (passage 9) cells. Scale bar, 100 μm. **B)** Measurement of nuclear area for control and patient fibroblasts at passages 13 and 11, respectively (mean ± s.e.m.). n > 100 nuclei/condition. **C)** Senescence assessment using the β-Galactosidase assay for control and patient fibroblasts at passages 9 and 6, respectively. **D)** Whole cell extracts of fibroblasts from control and patient at passages 10 and 14 (P10, P14) were analysed by western blot using antibodies against LA/C, LB1 or GAPDH. ECL signals were then scanned and quantified. The graph illustrates LA and LC ECL signal intensities as relative optical densities (O.D.) normalised versus GAPDH (n = 4) (mean ± s.e.m.). **E)** Human skin fibroblasts from control (passage 13) and patient (passage 11) were fixed, labelled with anti-LA/C or anti-LB1 antibodies, and observed by immunofluorescence microscopy. DNA was stained with Hoechst. Shown are images representative for normal nuclear morphology (Normal), nuclear dysmorphy (Dysm.) and lamina with a honeycomb pattern (Hon.). Asterisks indicate the honeycomb aspect of the lamin A/C network. Arrows indicate the regions with weak lamin B1 staining. The percentages of the different phenotypes are indicated on the pictures; n > 300 nuclei/condition. Scale bar, 10 μm.

To understand the molecular features of this novel mutation, we cultured patient fibroblasts. In comparison to the controls, patient skin fibroblasts were difficult to expand ex-vivo due to their slow growth. Slow growth was due to cells prematurely entering into senescence, demonstrated by their altered morphology (flattened and enlarged cells, greater nuclear area) (Fig 1A and 1B), the increased percentage of cells positive for senescence-associated β-galactosidase activity (Fig 1C) and the progressive decreased expression of lamin B1 (Fig 1D; [28]). Of note, similar levels of lamin A but a reduced level of lamin C were detected by immunoblot in patient versus control fibroblasts (Fig 1D). In the majority of control and patient fibroblasts, nuclei were ovoid and lamin A/C distributed normally at the nuclear envelope (NE) and in the nucleoplasm (Fig 1E). However, in ~4% of the patient cells, but in none of the control cells, (Fig 1E) the lamina network formed honeycomb-like structures stained for lamin A/C but locally depleted of lamin B1. Thus, the patient fibroblasts show specific defects in lamina organisation and cell proliferation capacity.

### The *LMNA* p.R388P mutation prevents the proper targeting of LA at the NE, which leads to the depletion of [emerin-LA] and [LAP2α-LA] complexes from the nuclear periphery

**Fig 2:**
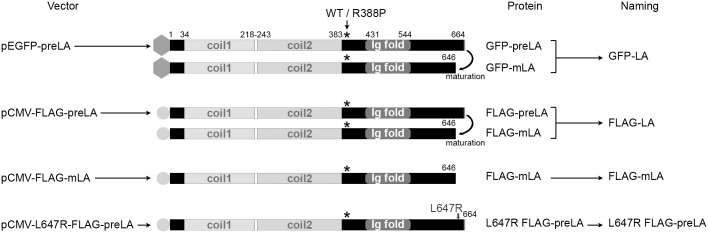
Schematic illustration of the secondary structures of A-type lamins expressed by transfection with constructs. Shown are prelamin A (preLA) and/or mature Lamin A (mLA) either WT or R388P, with or without the L647R mutation tagged with the FLAG (circles) or GFP sequences (hexagons) when using the pCMV-tag2A or pEGFP vectors, respectively. Stars indicate the position of the mutation R388P. Of note, the L647R mutation disrupts the proteolytic cleavage site of prelamin A, inhibiting the maturation of preLA into mLA.

To study the consequences of the L-CMD-related p.R388P mutation in myoblasts, C2C12 cells were transfected with pCMV plasmids encoding WT or R388P FLAG-tagged prelamin A (Fig 2). Transfection with a progeroid disorder-associated mutant L647R preLA [15], which cannot be processed into mature LA (mLA) due to a mutation in the Zmpste 24 proteolysis site, was used as a control of preLA expression (Fig 2).

**Fig 3:**
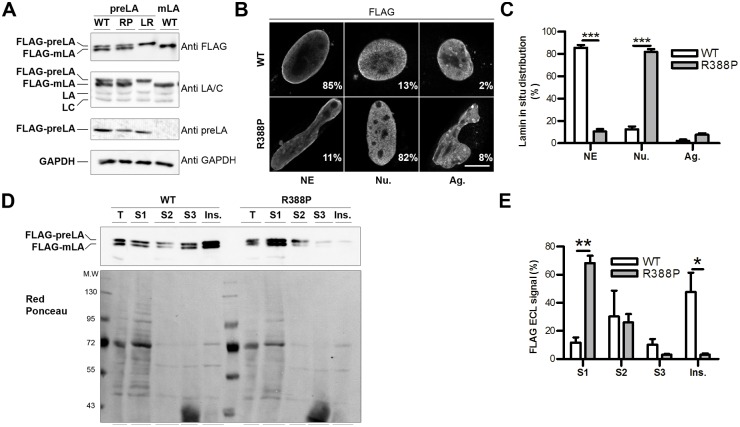
p.R388P mutation alters solubility and nuclear envelope targeting of lamin A. **A)** Whole cell extracts of C2C12 cells overexpressing wild-type (WT), R388P (RP), L647R (LR) FLAG-LA or WT FLAG-mature LA (mLA) were analysed by western blot using anti-FLAG, anti-LA/C (which recognise ectopic and endogenous A-type lamins), anti-prelamin A (preLA) and anti-GAPDH antibodies. **B)** C2C12 cells overexpressing WT or R388P FLAG-LA were fixed, labelled with anti-FLAG antibodies, and immunofluorescence observed at the confocal microscope. Representative cells presenting either a strong FLAG-lamin A staining at the nuclear envelope (NE), a predominant nucleoplasmic staining (Nu.) or the presence of small aggregates with ring—like structures (Ag.) are shown. Scale bar, 10 μm. **C)** Percentage of the three phenotypes NE, Nu. and Ag. for cells expressing WT or R388P FLAG-LA, as indicated. Data are the mean ± s.e.m. of 5 independent experiments and 100–250 nuclei/condition, *** p < 0.001 (two-way ANOVA with Bonferroni post-tests). **D)** C2C12 cells overexpressing WT or R388P FLAG-LA underwent cell fractionation to enrich the insoluble nuclear matrix intermediate filament scaffold. Proteins from soluble (S1, S2, S3) and insoluble (Ins.) fractions as well as whole cell extracts (T) were analysed in parallel by western blot using anti-FLAG antibodies. Samples loaded in each lane correspond to an identical cell number. Red Ponceau staining of nitrocellulose membrane illustrates the protein specificity of each fraction. **E)** ECL signals obtained in **D)** were scanned and quantified. The graph illustrates the distribution of FLAG tagged proteins into the different fractions assuming that 100% of the signal correspond to the total of signals recovered in [S1 + S2 + S3 + Ins.]. Data are the mean ± s.e.m. of 3 independent experiments. ** p < 0.01 and * p < 0.05 (two-way ANOVA with Bonferroni post-tests).

In transfected cells, both WT and R388P preLA proteins were incompletely processed as assessed by western blot comparing bands stained by anti-FLAG, anti-LA/C antibodies (which recognises both endogenous and ectopic lamins) and anti-prelamin A specific antibodies (Fig 3A). The mix of preLA and mLA is referred throughout our study as LA. Notably, R388P FLAG-LA was expressed at similar levels as WT FLAG-LA, likely reflecting the same stability of mutant and WT proteins. In situ, FLAG-LA was abnormally restricted to the nucleoplasm in 82% of cells expressing R388P-LA versus 13% of cells expressing WT-LA (Fig 3B and 3C). We also observed in a minority of cells (2–8%) expressing either WT or mutant LA, nuclear ring-like structures enriched in LA (Fig 3B and 3C). Concomitant to the predominant nucleoplasmic localisation of R388P-LA, cell fractionation revealed its greater solubilisation (68% of FLAG-LA R388P vs 12% of FLAG-LA WT in the surpernatant S1), and weaker integration into the nuclear lamina network (3% of FLAG-LA R388P vs 48% of FLAG-LA WT in the insoluble fraction) (Fig 3D and 3E).

**Fig 4:**
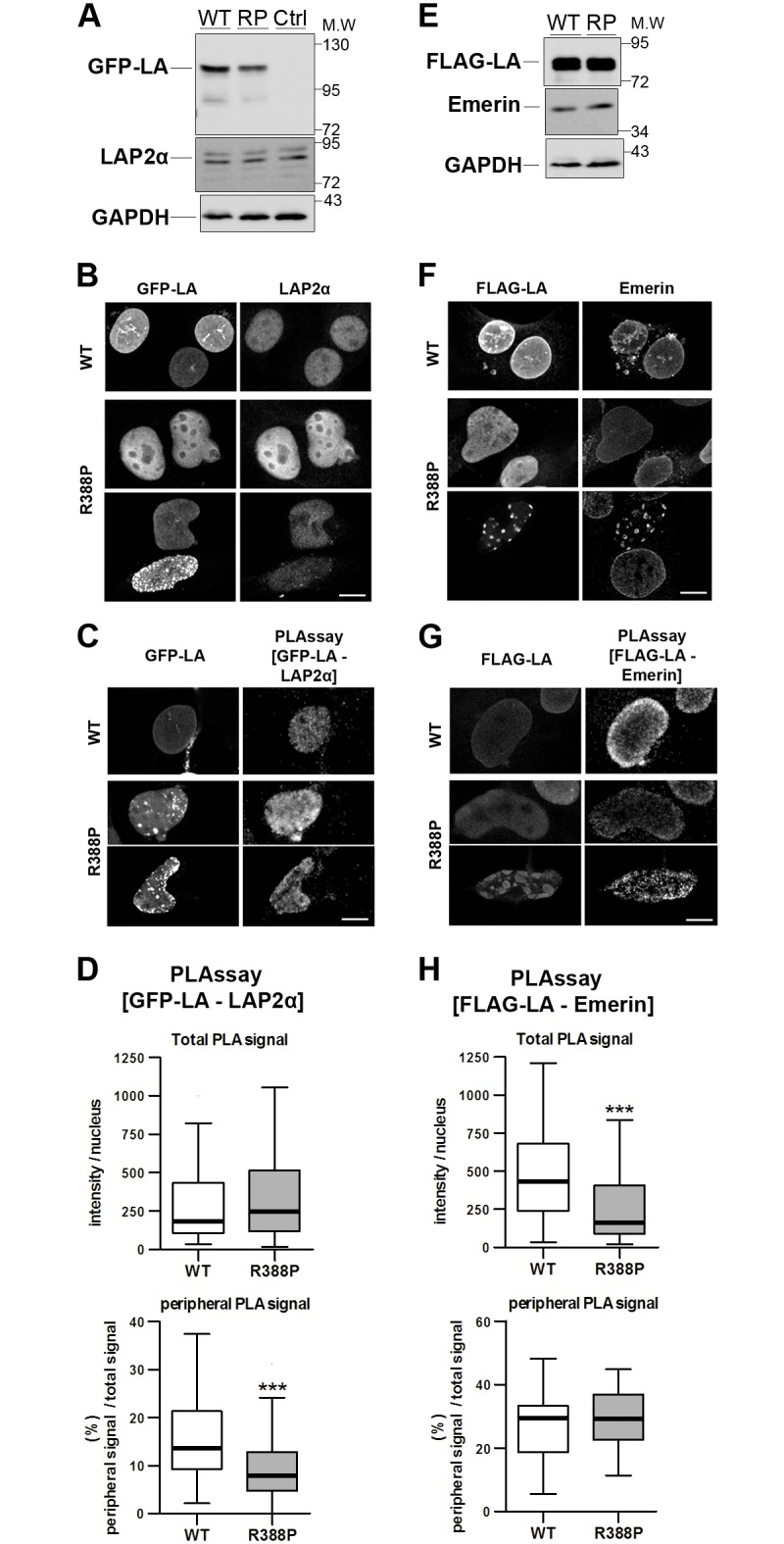
Changes in the lamin A in situ proximity with LAP2α and emerin in response to R388P-LA expression. **A,E)** Whole cell extracts of C2C12 cells either control (Ctrl) or overexpressing wild-type (WT) or R388P (RP) LA, tagged with GFP (left panel) or FLAG (right panel) were analysed by western blot using anti-GFP, anti-FLAG, anti-LAP2α, anti-emerin and anti-GAPDH antibodies, as indicated. **B-D, F-H)** C2C12 cells overexpressing WT (upper panel) or R388P (lower panels) GFP-LA or FLAG-LA were fixed, labelled with anti-GFP and anti-LAP2α or anti-FLAG and anti-emerin antibodies, and processed either for immunofluorescence **(B,F)** or proximity ligation assay (PLAssay) **(C,G)**, before observation at the confocal microscope. Scale bar, 10 μm. **D,H)** Quantification of PLA signals per nucleus among GFP/PLA or FLAG/LA positive cells as shown in **C,G)**. The graphs show the median intensity of the total PLA signals detected per nucleus (upper panel) and the median frequency of the PLA signals detected at the intranuclear periphery (lower panel). Boxes show first and third quartiles, bars are put according to Tukey method for n = 59 (WT) and 31 (R388P) nuclei for **D** and n = 67 (WT) and 74 (R388P) nuclei for **H** *** p < 0.001 (Mann Whitney test).

This suggested that the p.R388P mutation might alter the binding of LA to specific partners at the nuclear periphery or within the nucleoplasm. We focused on two partners that bind LA predominantly within the nucleoplasm (LAP2α, Fig 4A and 4B) or at the NE (emerin Fig 4E and 4F), and quantified the occurrence of complexes formed between ectopic LA and endogenous LAP2α or emerin, using proximity ligation assays (PLA). PLA signals corresponding to [LAP2α—GFP-LA] complexes, of similar total amount in myoblasts expressing WT or R388P GFP-LA, were mainly detected throughout the nucleoplasm (Fig 4C and 4D). However, the [LAP2α—GFP-LA] complexes localised at the NE were significantly less frequent in nuclei expressing R388P versus WT GFP-LA (9% vs 16%, respectively, Fig 4D). Quantification of PLA signals related to [FLAG-LA—emerin] complexes revealed a significant decrease (from 100 to 55%) in their global amount per nucleus in cells expressing R388P versus WT FLAG-LA (Fig 4G and 4H), while their subnuclear distribution was unchanged.

Therefore, expression of R388P-LA triggers a significant depletion of LA interaction with both LAP2α and emerin specifically at the intranuclear periphery.

### Nuclear dysmorphy induced by R388P LA is not due to preLA accumulation

**Fig 5:**
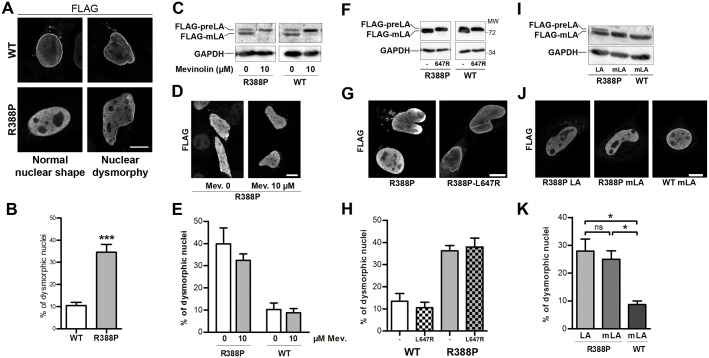
Different maturation intermediates of R388P prelamin A induce nuclear dysmorphy. **A, D, G** and **J**, immunofluorescently-labelled cells observed under confocal microscopy; scale bars, 10 μm. **A)** C2C12 cells overexpressing WT (WT) or mutant (R388P) FLAG-LA were fixed and labelled with anti-FLAG antibodies. Representative nuclei with a normal shape (ovoid) or with dysmorphy are shown. **B)** Percentage of dysmorphic nuclei among cells expressing either WT FLAG-LA or R388P FLAG-LA, as evaluated by visual observation (mean ±s.e.m.), *** p < 0.001 for n = 8 independent experiments with >100 nuclei/experiment (Mann Whitney test). **C)** Whole cell extracts of C2C12 cells overexpressing WT or R388P FLAG-LA and treated with DMSO (0) or 10 μM (10) Mevinolin (Mev) for 18 h, were analysed by western blot using anti-FLAG antibodies. GAPDH was used as a loading control. **D)** C2C12 cells as described in **C)** were fixed and labelled with anti-FLAG antibodies. **E)** Percentage of dysmorphic nuclei of cell populations assessed by visual observation as in **D)** (mean ± s.e.m.), for n = 3 independent experiments and >100 nuclei/experiment (Kruskal-Wallis test with pairwise comparisons of groups). **F)** Whole cell extracts of C2C12 cells overexpressing FLAG-tagged R388P LA, R388P-L647R preLA, WT LA or L647R preLA were analysed by western blot using anti-FLAG and anti-GAPDH antibodies. **G)** C2C12 cells as described in **F)** were fixed, labelled with anti-FLAG antibodies. **H)** Percentage of dysmorphic nuclei for cell populations observed in **G)** (mean ± s.e.m.), p = 0.9 for n = 5 independent experiments with cells expressing R388P FLAG-LA and >100 nuclei/experiment (Mann Whitney test). n = 2 independent experiments with cells expressing R388P FLAG-LA and >100 nuclei/experiment. **I)** Whole cell extracts of C2C12 cells overexpressing R388P FLAG-LA, R388P FLAG-mLA or WT FLAG-mLA were analysed by western blot using anti-FLAG and anti-GAPDH antibodies. **J)** C2C12 cells as described in **I)** were fixed and labelled with anti-FLAG antibodies. **K)** Percentage of dysmorphic nuclei for cell populations observed as in **J)** (mean ± s.e.m.), * p < 0.05 for n = 4 independent experiments and >100 nuclei/experiment (Kruskal-Wallis test with pairwise comparisons of groups).

Nuclear shape was assessed on cells stained for DNA and FLAG-LA by observation (Fig 5A) and by measurement of the mean nuclear circularity (A and B in S2 Fig). Expression of R388P vs WT FLAG-LA induced, i) a 3.5-fold increase in dysmorphic nuclei (from 11 to 35%) with a decrease in the mean nuclear circularity (from 0.82 to 0.69) (Fig 5B and A and B in S2 Fig) and ii) an increase in the severity of dysmorphies, as shown by the decreased nuclear circularity in the subpopulation of dysmorphic nuclei (from 0.71 to 0.60; C in S2 Fig). To decipher whether nuclear dysmorphy was related to a preLA processing defect, we forced the accumulation of preLA by incubating cells with mevinolin, an HMG-CoA reductase inhibitor that decreases the synthesis of farnesyl pyrophosphate, the substrate of farnesyl transferases (Fig 5C–5E). Separately, we overexpressed the double mutant R388P-L647R FLAG-preLA, which can undergo farnesylation but not the proteolytic maturation needed for the synthesis of mLA (Fig 5F–5H). While the treatment of cells with 10 μM mevinolin blocked preLA processing as expected (Fig 5C), it did not modify its subnuclear distribution (Fig 5D) or changed the frequency of nuclear dysmorphy in cells expressing R388P or WT FLAG-LA (40% vs 32%, and 10 vs 9%, respectively; Fig 5E). The R388P-L647R double mutant FLAG-preLA, which remained constitutively farnesylated (Fig 5F) was detected at the NE (Fig 5G) and induced a frequency of dysmorphic nuclei similar to the R388P FLAG-LA (38% vs 36%; Fig 5H). Additionally, the mutant mature lamin A (R388P-mLA) localised exclusively within the nucleoplasm (Fig 5I and 5J) and induced a frequency of nuclear dysmorphy similar to R388P-LA (25% vs 28%; Fig 5K).

In conclusion, nuclear dysmorphy induced by R388P lamins A in myoblasts, does not rely on defective prelamin A processing.

### Releasing of forces exerted on nuclei by either the cytoskeleton or chromatin does not rescue nuclear shape in myoblasts expressing R388P LA

Considering the hypothetical models proposed to generate dysmorphic nuclei (see Introduction), we investigated whether nuclear dysmorphy induced by R388P-LA could be reversed by releasing forces exerted by different elements of the cytoskeleton, including microtubules, actin filaments and intermediate filaments (as desmin) or by modifying the degree of chromatin compaction.

**Fig 6:**
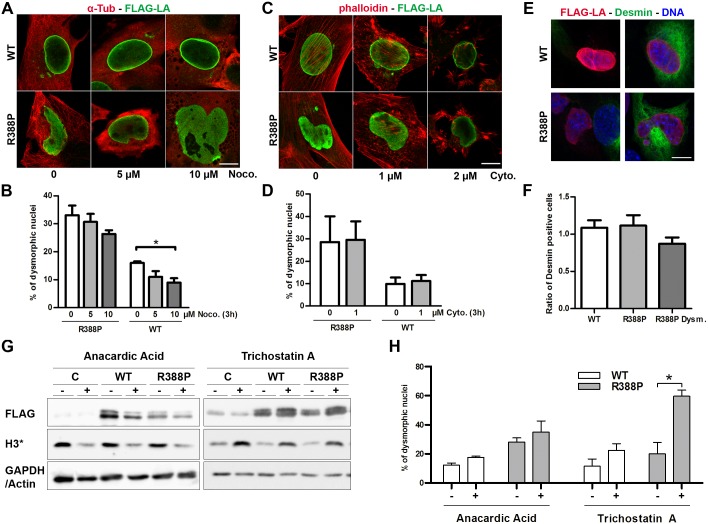
Altering cytoskeletal element integrity or global histone acetylation pattern does not rescue nuclear shape in myoblasts expressing R388P LA. In **A, C** and **E**, immunofluorescently-labelled cells were observed under confocal microscopy; scale bars, 10 μm. **A)** C2C12 cells overexpressing WT or R388P FLAG-LA were treated with 0.05% DMSO (0) or with 5 to 10 μM Nocodazole (Noco.) for 3 h at 37°C. After fixation, cells were labelled with rabbit anti-FLAG (green) and mouse anti-α-tubulin antibodies (red). **B)** The graph illustrates the percentage (mean ± s.e.m.) of dysmorphic nuclei among cells treated as shown in **A)**, * p < 0.05 for n = 3 independent experiments and >100 nuclei/experiment (WT and R388P data were analysed separately by Kruskal-Wallis tests with pairwise comparisons of groups). **C)** C2C12 cells overexpressing WT or R388P FLAG-LA were treated with 0.2% DMSO (0) or with the actin depolymerising agent Cytochalasin D at 1 or 2 μM (Cyto.) for 3 h at 37°C. After fixation, cells were labelled with rabbit anti-FLAG antibodies and with phalloidin. **D)** Percentage (mean ± s.e.m.) of dysmorphic nuclei among cells treated as in **C)**, p>0.05 for n = 3 independent experiments and >100 nuclei/experiment (WT and R388P data were analysed separately by Kruskal-Wallis tests with the pairwise comparisons of groups). **E)** C2C12 cells overexpressing WT or R388P FLAG-LA were fixed and labelled with rabbit anti-FLAG (red) and mouse anti-desmin (green) antibodies. DNA was stained with Hoechst (blue). Representative cells illustrating nuclear shape in the absence or presence of endogenous desmin expression (left and right panels, respectively). **F)** The graph illustrates the mean ratio (± s.e.m.) of desmin-positive vs -negative cells for transfected cells versus untransfected control cells. Are considered the whole population of cells expressing WT or mutant (R388P) FLAG-LA and the subpopulation of cells expressing R388P FLAG-LA with dysmorphic nuclei (R388P Dysm.), p>0.05 for n = 4 independent experiments and >100 nuclei/experiment (Kruskal-Wallis tests with pairwise comparisons of groups). **G)** C2C12 cells, control (C) or expressing WT or R388P FLAG-LA were incubated in the absence (-) or presence (+) of anacardic acid (100 μM) or trichostatin A (100 nM), as indicated. Whole cell extracts were analysed by western blot using anti-FLAG, anti-H3K9ac (left panel) and anti-H3K27ac (right panel) antibodies. GAPDH and Actin detection were used as loading controls in the left and right panels, respectively. **H)** C2C12 cells expressing WT or R388P FLAG-LA were treated with drugs as in **G)**. The graph illustrates the percentage of dysmorphic nuclei for cell populations (mean ± s.e.m), * p < 0.05 for n = 3 independent experiments and >100 nuclei/experiment (Kruskal-Wallis test with pairwise comparisons of groups).

Depolymerisation of microtubules with nocodazole did not modify significantly the frequency of dysmorphic nuclei in myoblasts expressing R388P-LA (Fig 6A and 6B). Conversely, nocodazole reduced by 44% the frequency of dysmorphic nuclei induced upon overexpression of WT-LA (Fig 6B). We then depolymerised the actin filament network using cytochalasin D. A high concentration of the drug (2 μM) induced the loss of cell adherence and shrinkage of nuclei, precluding the measurement of nuclear dysmorphy frequency (Fig 6C). However, at 1 μM, cytochalasin D significantly disrupted the actin network, but it did not modify the frequency of nuclear dysmorphies in myoblasts expressing R388P-LA (29 vs 30% of dysmorphic nuclei; Fig 6C and 6D). The frequency of desmin-positive cells was similar in cells expressing WT or R388P FLAG-LA, as well as in the subpopulation of myoblasts with dysmorphic nuclei caused by R388P-LA expression (Fig 6E and 6F). In conclusion, disruption of one or other cytoskeletal elements does not restore nuclear shape in myoblasts expressing R388P-LA.

Using antibodies directed against H3K9ac or H3K27ac, we observed an increased histone acetylation upon R388P-LA overexpression in C2C12 myoblasts, suggesting that the mutation could alter some chromatin epigenetic marks (A and B in S3 Fig). Drugs affecting chromatin organisation by inhibiting histone deacetylase (HDACs) or the specific histone acetyl transferase HAT named NAT10 have been shown to decrease the frequency of nuclear dysmorphies induced by progerin expression or lamin A/C depletion [17, 29]. Here, we tested the impact of anacardic acid, an HAT inhibitor and of trichostatin A (TSA), inhibitor of HDACs I and II. Although anacardic acid induced a global decrease in H3K9 acetylation as expected, it did not rescue nuclear dysmorphy (Fig 6G and 6H). Moreover, although TSA induced a global increase in H3K27 acetylation as expected, it increased nuclear dysmorphy specifically in cells expressing R388P FLAG-LA (Fig 6G and 6H) but not in cells expressing WT FLAG-LA. These observations further highlight the extreme sensitivity of nuclei to deform in response to variations in the chromatin compaction state, specifically in the context of R388P-LA mutation.

## Discussion

### The R388P *LMNA* mutation is associated with a rare and severe phenotype associating congenital muscular dystrophy and lipodystrophy

To our knowledge, the association of L-CMD with lipodystrophy has not been described. The few *LMNA* mutations (~23) previously reported as responsible for L-CMD are located all along the different regions of A-type lamins (N-ter, coil 1a, coil2 and C-ter) [30–33], whereas mutations responsible for lipodystrophy predominantly affect the C-ter domain [34]. Only one mutation of the 388^th^ amino acid of lamin A was previously shown to be pathogenic, and was associated with L-CMD (R388C, [35]).

Arginine 388 belongs to a group of residues (E358, M371, L387, R388) located at the end of coil 2B and at the C-ter of the rod domain that are highly conserved between species from human to *Xenopus* (S1 Fig). Interestingly, we previously described the p.L387V mutation as responsible for a metabolic syndrome associated with muscular signs [34]. Based on crystal structures of lamin A coil 2B dimer, these amino acids were proposed to play a key role in the longitudinal head to tail association of lamins (S4 Fig; [36]). Accordingly, R388P (this study) as well as R386K, E358K and M371K mutations [37] impede proper formation of a lamina lattice at the NE of myoblasts. The *LMNA* p.R388P and E358K mutations can cause L-CMD, whereas the two other *LMNA* mutations are responsible for the less severe phenotype of Emery-Dreifuss muscular dystrophy. Thus, we speculated that, in addition to altering the lamin assembly properties with expected consequences on viscosity properties of nuclei [38], the R388P mutation might impair other important functions of lamin A. In that setting, the observed reduction in the amount of [lamin A—emerin] complexes at the NE and of [lamin A—LAP2α] complexes at the intranuclear periphery might trigger an inappropriate modulation of specific cellular pathways known to be regulated by emerin and LAP2α in muscle, including those mediated by BAF, involved in higher order structure of chromatin [39, 40]. In addition, the increased global acetylation of histone H3 might reflect additional effects of the R388P lamin A mutant on chromatin organisation and gene expression. In agreement, WT-LA was shown to bind a subset of genomic regions including promoter regions of genes related to development, whose identity depends on the differentiation state of the cells [41]. We thus propose that the mutant R388P-LA alters the peripheral heterochromatin anchorage, the global 3D-structure of the genome and gene expression, with drastic consequences on the fate of cells related to skeletal muscle and adipose tissues.

### Specificity of the mechanisms underlying the abnormal phenotypes triggered in myoblasts by R388P-LA

Since the discovery of *LMNA* mutations as responsible for several diseases, extensive work has been produced to characterise the associated cellular phenotypes. However, only few studies have been performed in the context of L-CMD [32, 42, 43]. The phenotypic features we observed in skin fibroblasts from the patient have also been reported in other laminopathies, including lipodystrophies, muscular dystrophies and premature ageing syndromes [7, 8, 11, 12, 13, 34, 44, 45, 46]. These features include the disorganisation of the nuclear lamina with a honeycomb pattern, the local nuclear depletion of lamin B1 and/or the premature entry into senescence. However, the reduced cell proliferation capacities of the mutated fibroblasts limited their use for further mechanistic studies.

To our knowledge, our study is the first to precisely characterise the phenotypes of myoblasts expressing a mutant lamin A responsible for L-CMD. Beside nuclear dysmorphies, previously associated with other laminopathies including Hutchinson-Gilford progeria, our study reveals several specific defects induced by the R388P-LA: i) R388P-LA accumulates in the nucleoplasm and is more soluble than WT-LA (this study), whereas progerin, which is constitutively farnesylated, forms a stiff lamina at the NE [47, 48]; ii) nuclear dysmorphy relies on defective prelamin A processing in HGPS and some lipodystrophies but not in the R388P context; iii) preventing lamin A farnesylation rescues nuclear shape in cells expressing progerin [19, 49, 50] but not R388P-LA; iv) releasing the forces exerted on the NE by the cytoskeleton rescues nuclear shape of fibroblasts from patients with progeria and from *LMNA*^-/-^ fibroblasts [17] but not of myoblasts expressing R388P-LA; v) chromatin remodelling with drugs such as the HDAC inhibitor TSA could reverse the nuclear dysmorphy in the progeria context [29], but it increased the frequency of dysmorphic nuclei caused by R388P-LA.

In conclusion, our study highlights the multiple deleterious impacts of the *LMNA* p.R388P mutation, responsible for a complex and severe multisystemic laminopathy phenotype, on the organisation of the nuclear compartment in myoblasts. The difficulty in reversing the abnormal cellular nuclear phenotypes using drugs validated in other laminopathy contexts highlights a need to develop combinatorial approaches or to consider the depletion of that mutant lamin A for therapeutic purposes.

## Supporting Information

S1 Fig Amino acid sequences alignment for lamin A/C across different species. The position of the mutation described in this study is shown in bold. The corresponding Swiss Prot accession numbers are P02545, P48679 and P48678 for human, rat and mouse lamin A/C, respectively, and P13648 and P11048 for chicken and *Xenopus laevis* lamin A, respectively.
(PDF)

S2 Fig Characteristics of dysmorphic nuclei, in terms of circularity and surface area. C2C12 cells overexpressing WT (WT) or mutant (R388P) FLAG-LA were fixed and labelled with anti-FLAG antibodies and analysed by immunofluorescence under confocal microscopy. **A)** Nuclear circularity (mean value ± s.e.m.) of whole cell populations expressing either WT FLAG-LA or R388P FLAG-LA. ** p = 0.002 for n = 6 independent experiments (Mann Whitney test) **B)** Percentage (mean ± s.e.m.) of dysmorphic nuclei among cells expressing either WT FLAG-LA or R388P FLAG-LA. The criteria of dysmorphy rely on the circularity value (inferior to 0.74) or on the visual observation, as indicated. ** p = 0.002 for n = 6 independent experiments (Mann Whitney test). **C**) Nuclear circularity (mean ± s.e.m.) of dysmorphic nuclei as revealed by visual observation of cell populations expressing WT FLAG-LA or R388P FLAG-LA. ** p = 0.002 for n = 6 independent experiments (Mann Whitney test). **D**) Size of nuclei (mean expressed in μm^2^ ± s.e.m.) with either normal shape (Norm.) or with dysmorphy (Dysm.) as assessed by visual observation of cell populations expressing either WT FLAG-LA or R388P FLAG-LA. * p = 0.01 for n = 6 independent experiments (Kruskal-Wallis test with the pairwise comparison of groups).
(PDF)

S3 Fig Increased H3K9ac acetylation detection in cells expressing R388P FLAG-LA. **A)** C2C12 cells overexpressing WT or R388P FLAG-LA were fixed and labelled with mouse anti-FLAG (green) and rabbit anti-H3K9ac (red) antibodies before observation under confocal microscopy. Arrows in A) indicate the absence of H3K9ac signal in cells overexpressing WT lamins A. Scale bar, 20 μm. **B)** The graph illustrates the H3K9ac median immunofluorescence signal intensity observed per nucleus of cells processed as in A) that express either WT or R388P-LA. Signals are normalised to the signal measured in untransfected cells for 3 independent experiments (1, 2, 3). Boxes show first and third quartiles, bars are put according to Tukey method for n > 125 nuclei per condition, *** p < 0.001 (Mann Whitney test).
(PDF)

S4 Fig Model for head-to-tail association of lamin A dimers, according to Strelkov et al. 2004 [36]. This model postulates electrostatic attraction of lamin A dimers via distinct positively charged patches (rich in arginine; blue) and negatively charged patches (rich in aspartic and glutamic acids; red). The position of the head, coil 1, coil 2 and C-ter regions and of the Ig-fold domain of A-type lamins are identified. Numbers refer to the amino acid sequence. The localisation of the p.R388P mutation is highlighted within one positively charged patch close to the end of coil 2.
(PDF)

S1 File Primary data. (XLSX)

## Abbreviations

a.a.: amino acids
CMD: congenital muscular dystrophy
L-CMD: *LMNA*-dependent congenital muscular dystrophy
mLA: mature lamin A
NE: nuclear envelope
preLA: prelamin A
