# Supplementary material for: A Novel Lamin A Mutant Responsible for Congenital Muscular Dystrophy Causes Distinct Abnormalities of the Cell Nucleus
Source: PLoS One. 2017 Jan 26;12(1):e0169189. doi: 10.1371/journal.pone.0169189 (PMC5268432; doi:10.1371/journal.pone.0169189)
Supplement: S1 Fig — The position of the mutation described in this study is shown in bold. The corresponding Swiss Prot accession numbers are P02545, P48679 and P48678 for human, rat and mouse lamin A/C, respectively, and P13648 and P11048 for chicken and Xenopus laevis lamin A, respectively. (PDF) [file pone.0169189.s001.pdf]

Barateau et al.

A novel lamin A mutant responsible for congenital muscular dystrophy causes distinct abnormalities of the cell nucleus

Organism	Protein	Sequence
	Coil 2	⟶ ⟵ Tail ⟶
		**R388**
Human	Lamin A/C	DEYKIHAYRKLLEGEEERL**R**LSPSPTSQRSRGRAS
Rat	Lamin A/C	DEYKIHAYRKLLEGEEERL**R**LSPSPTSQRSRGRAS
Mouse	Lamin A/C	DEYKIHAYRKLLEGEEERL**R**LSPSPTSQRSRGRAS
Chicken	Lamin A	DEYKINAYRKLLEGEEERL**R**LSPSPSSQRGARSSG
Xenopus laevis	Lamin A	DEYKINAYRKLLEGEEERL**R**LSPSPNTQKRSARTI

S1 Fig. Amino acid sequences alignment for lamin A/C across different species. The position of the mutation described in this study is shown in bold. The corresponding Swiss Prot accession numbers are P02545, P48679 and P48678 for human, rat and mouse lamin A/C, respectively, and P13648 and P11048 for chicken and *Xenopus laevis* lamin A, respectively.
