# Supplementary material for: A Novel Lamin A Mutant Responsible for Congenital Muscular Dystrophy Causes Distinct Abnormalities of the Cell Nucleus
Source: PLoS One. 2017 Jan 26;12(1):e0169189. doi: 10.1371/journal.pone.0169189 (PMC5268432; doi:10.1371/journal.pone.0169189)
Supplement: S2 Fig — C2C12 cells overexpressing WT (WT) or mutant (R388P) FLAG-LA were fixed and labelled with anti-FLAG antibodies and analysed by immunofluorescence under confocal microscopy. A) Nuclear circularity (mean value ± s.e.m.) of whole cell populations expressing either WT FLAG-LA or R388P FLAG-LA. ** p = 0.002 for n = 6 independent experiments (Mann Whitney test) B) Percentage (mean ± s.e.m.) of dysmorphic nuclei among cells expressing either WT FLAG-LA or R388P FLAG-LA. The criteria of dysmorphy rely on the circularity value (inferior to 0.74) or on the visual observation, as indicated. ** p = 0.002 for n = 6 independent experiments (Mann Whitney test). C) Nuclear circularity (mean ± s.e.m.) of dysmorphic nuclei as revealed by visual observation of cell populations expressing WT FLAG-LA or R388P FLAG-LA. ** p = 0.002 for n = 6 independent experiments (Mann Whitney test). D) Size of nuclei (mean expressed in μm2 ± s.e.m.) with either normal shape (Norm.) or with dysmorphy (Dysm.) as assessed by visual observation of cell populations expressing either WT FLAG-LA or R388P FLAG-LA. * p = 0.01 for n = 6 independent experiments (Kruskal-Wallis test with the pairwise comparison of groups). (PDF) [file pone.0169189.s002.pdf]

Barateau et al.

A novel lamin A mutant responsible for congenital muscular dystrophy causes distinct abnormalities of the cell nucleus

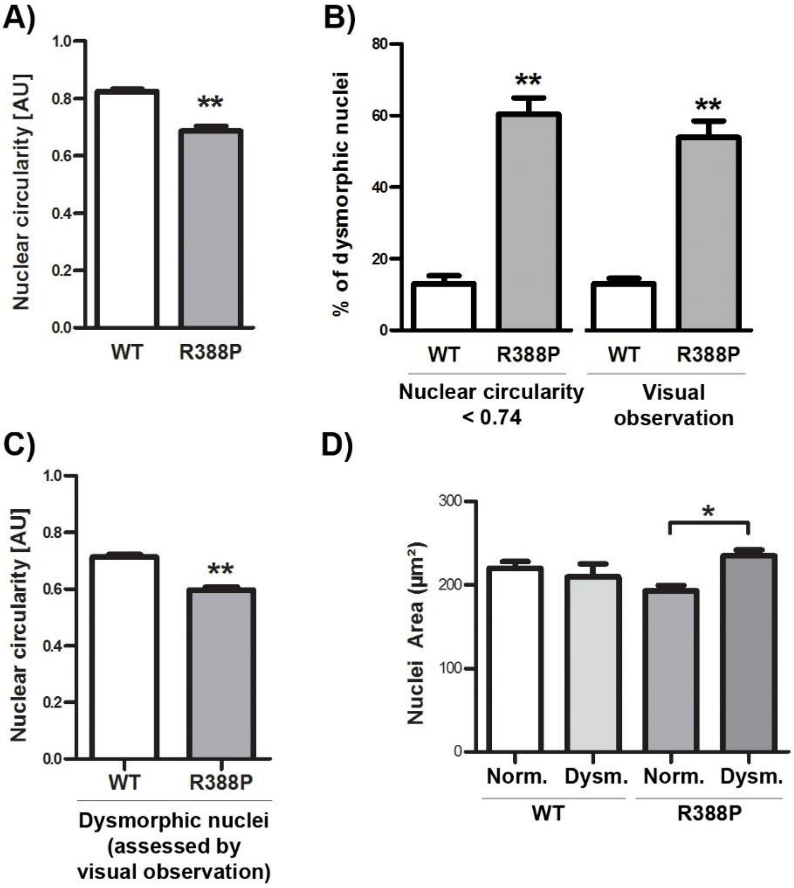

S2 Fig. Characteristics of dysmorphic nuclei, in terms of circularity and surface area. C2C12 cells overexpressing WT (WT) or mutant (R388P) FLAG-LA were fixed and labelled with anti-FLAG antibodies and analysed by immunofluorescence under confocal microscopy. **A)** Nuclear circularity (mean value ± s.e.m.) of whole cell populations expressing either WT FLAG-LA or R388P FLAG-LA. ** p = 0.002 for n = 6 independent experiments (Mann Whitney test) **B)** Percentage (mean ± s.e.m.) of dysmorphic nuclei among cells expressing either WT FLAG-LA or R388P FLAG-LA. The criteria of dysmorphy rely on the circularity value (inferior to 0.74) or on the visual observation, as indicated. ** p = 0.002 for n = 6 independent experiments (Mann Whitney test). **C**) Nuclear circularity (mean ± s.e.m.) of dysmorphic nuclei as revealed by visual observation of cell populations expressing WT FLAG-LA or R388P FLAG-LA. ** p = 0.002 for n = 6 independent experiments (Mann Whitney test). **D**) Size of nuclei (mean expressed in µm² ± s.e.m.) with either normal shape (Norm.) or with dysmorphy (Dysm.) as assessed by visual observation of cell populations

expressing either WT FLAG-LA or R388P FLAG-LA. * $p = 0.01$ for $n = 6$ independent experiments (Kruskal-Wallis test with the pairwise comparison of groups).
