# Supplementary material for: A Novel Lamin A Mutant Responsible for Congenital Muscular Dystrophy Causes Distinct Abnormalities of the Cell Nucleus
Source: PLoS One. 2017 Jan 26;12(1):e0169189. doi: 10.1371/journal.pone.0169189 (PMC5268432; doi:10.1371/journal.pone.0169189)
Supplement: S3 Fig — A) C2C12 cells overexpressing WT or R388P FLAG-LA were fixed and labelled with mouse anti-FLAG (green) and rabbit anti-H3K9ac (red) antibodies before observation under confocal microscopy. Arrows in A) indicate the absence of H3K9ac signal in cells overexpressing WT lamins A. Scale bar, 20 μm. B) The graph illustrates the H3K9ac median immunofluorescence signal intensity observed per nucleus of cells processed as in A) that express either WT or R388P-LA. Signals are normalised to the signal measured in untransfected cells for 3 independent experiments (1, 2, 3). Boxes show first and third quartiles, bars are put according to Tukey method for n > 125 nuclei per condition, *** p < 0.001 (Mann Whitney test). (PDF) [file pone.0169189.s003.pdf]

Barateau et al.

A novel lamin A mutant responsible for congenital muscular dystrophy causes distinct abnormalities of the cell nucleus

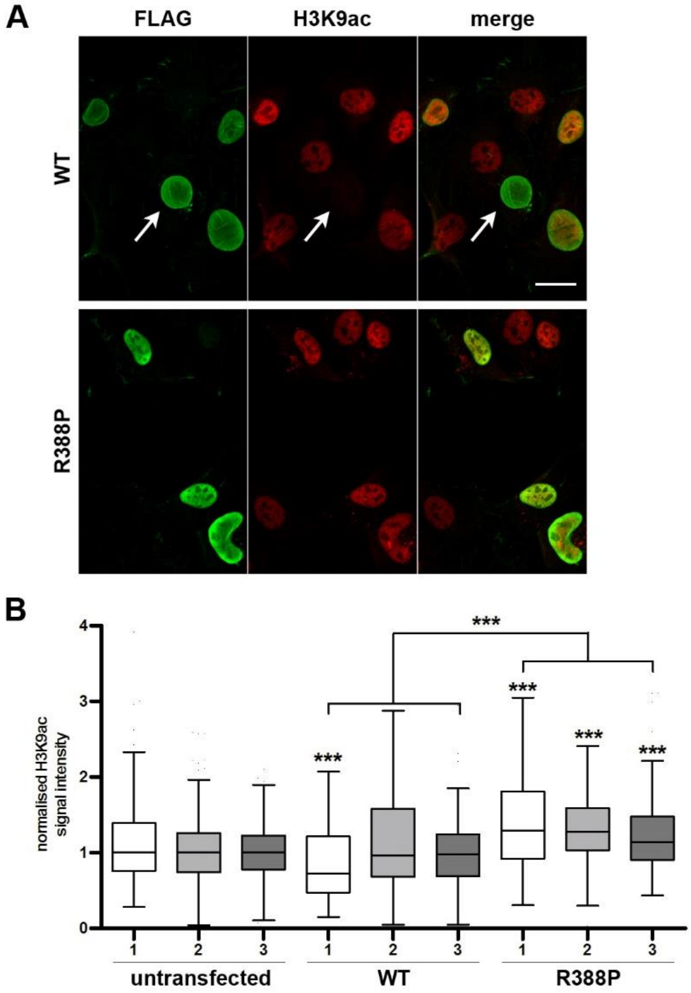

S3 Fig. Increased H3K9ac acetylation detection in cells expressing R388P FLAG-LA.
A) C2C12 cells overexpressing WT or R388P FLAG-LA were fixed and labelled with mouse anti-FLAG (green) and rabbit anti-H3K9ac (red) antibodies before observation under confocal microscopy. Arrows in A) indicate the absence of H3K9ac signal in cells overexpressing WT lamins A. Scale bar, 20 µm. **B)** The graph illustrates the H3K9ac median immunofluorescence signal intensity observed per nucleus of cells processed as in A) that express either WT or R388P-LA. Signals are normalised to the signal measured in untransfected cells for 3 independent experiments (1, 2, 3). Boxes show first and third quartiles, bars are put according to Tukey method for n > 125 nuclei per condition, *** $p < 0.001$ (Mann Whitney test).
