# Supplementary material for: A Novel Lamin A Mutant Responsible for Congenital Muscular Dystrophy Causes Distinct Abnormalities of the Cell Nucleus
Source: PLoS One. 2017 Jan 26;12(1):e0169189. doi: 10.1371/journal.pone.0169189 (PMC5268432; doi:10.1371/journal.pone.0169189)
Supplement: S4 Fig — This model postulates electrostatic attraction of lamin A dimers via distinct positively charged patches (rich in arginine; blue) and negatively charged patches (rich in aspartic and glutamic acids; red). The position of the head, coil 1, coil 2 and C-ter regions and of the Ig-fold domain of A-type lamins are identified. Numbers refer to the amino acid sequence. The localisation of the p.R388P mutation is highlighted within one positively charged patch close to the end of coil 2. (PDF) [file pone.0169189.s004.pdf]

Barateau et al.

A novel lamin A mutant responsible for congenital muscular dystrophy causes distinct abnormalities of the cell nucleus

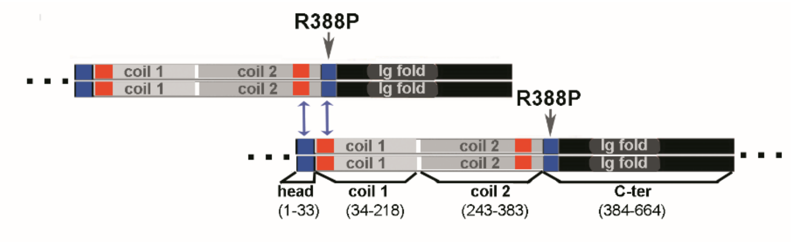

S4 Fig. Model for head-to-tail association of lamin A dimers, according to Strelkov et al. 2004 [36]. This model postulates electrostatic attraction of lamin A dimers via distinct positively charged patches (rich in arginine; blue) and negatively charged patches (rich in aspartic and glutamic acids; red). The position of the head, coil 1, coil 2 and C-ter regions and of the Ig-fold domain of A-type lamins are identified. Numbers refer to the amino acid sequence. The localisation of the p.R388P mutation is highlighted within one positively charged patch close to the end of coil 2.
